# Non-Invasive Assessment of Hypertonic Muscle Properties After Botulinum Toxin Neuromodulation in Post-Stroke Patients: A Systematic Literature Review of Recent Evidence (2023–2025) on Mobility and Balance

**DOI:** 10.3390/life16071120

**Published:** 2026-07-05

**Authors:** Sebastian Giuvara, Gelu Onose, Constantin Munteanu, Cristina Popescu, Aura Spinu, Andrada Mirea, Aurelian Anghelescu

**Affiliations:** 1 Faculty of Medicine, University of Medicine and Pharmacy “Carol Davila”, 020021 Bucharest, Romania; sebastian.giuvara@drd.umfcd.ro (S.G.); gelu.onose@umfcd.ro (G.O.); andrada.mirea@umfcd.ro (A.M.); aurelian.anghelescu@umfcd.ro (A.A.); 2 The Neuromuscular Rehabilitation Clinic Division, Teaching Emergency Hospital “Bagdasar-Arseni”, 041915 Bucharest, Romania; constantin.munteanu.biolog@umfiasi.ro; 3 Faculty of Medical Bioengineering, University of Medicine and Pharmacy “Grigore T. Popa” Iasi, 700454 Iasi, Romania; 4 National Teaching Center for Children’s Neurorehabilitation “Dr. Nicolae Robanescu”, 041408 Bucharest, Romania

**Keywords:** post-stroke spasticity, post-stroke rehabilitation, viscoelastic muscle properties, balance, stabilometry

## Abstract

Background: Post-stroke spasticity is a frequent and disabling consequence of stroke, including when affecting the lower limbs, where it may impair stance, gait, balance, postural control, functional independence and quality of life. Botulinum toxin type A (BoNT-A) is widely used as a focal neuromodulatory treatment for post-stroke spasticity. However, the relationship between BoNT-A-induced reduction in muscle hypertonia, objective changes in spastic muscle’s biomechanical properties, and functional outcomes such as mobility and balance remains insufficiently clarified. This systematic review aimed to synthesize recent evidence regarding the non-invasive assessment of spastic muscle properties following BoNT-A administration in post-stroke patients, with emphasis on mobility and balance outcomes. Methods: A systematic literature review was conducted according to the Preferred Reporting Items for Systematic Reviews and Meta-Analyses (PRISMA) guidelines. The search was performed in international electronic databases and included studies published between 1 January 2023 and 31 December 2025. The search strategy used specific keywords and keyword combinations/syntaxes, contextually, related to the topic of interest. Results: A total of 32 studies met the eligibility criteria and were included in the final data analysis and synthesis, comprising 13 primary clinical studies—6 randomized or controlled interventional studies and 7 observational studies—together with 12 reviews or evidence syntheses, 3 technical or clinical framework papers, and 4 survey, epidemiological, health-services or health-economic studies. Overall, the included articles addressed BoNT-A treatment in post-stroke spasticity, with partial focus on muscle properties, gait, mobility, and functional outcomes. However, only a limited number of studies investigated objective non-invasive assessment methods, and few directly related muscle-property changes in balance and mobility outcomes. Formal risk-of-bias assessment and quantitative synthesis were not performed because of the substantial heterogeneity of the included evidence, with only two studies being potentially suitable for pooling and these addressing different muscle groups, interventions, and outcome domains. Discussion and Conclusions: The reviewed literature confirms the clinical relevance of BoNT-A in the management of post-stroke spasticity. However, most studies assess treatment effects mainly through clinical scales, while objective evaluation of muscle stiffness, elasticity, viscoelastic properties, and their relationship with mobility and balance remains limited. Although some studies address gait, functional recovery, or muscle-related changes, the combined use of BoNT-A treatment, myotonometric assessment, and proprioceptive–stabilometric evaluation is largely absent. Therefore, current evidence highlights an important research gap and supports the need for future longitudinal studies integrating non-invasive biomechanical and balance assessment tools to better monitor treatment response and guide individualized neurorehabilitation in post-stroke patients.

## 1. Introduction

Stroke remains one of the leading causes of long-term disability worldwide and represents a major public health burden due to its high incidence/prevalence frequency, mortality, and persistent functional sequelae [1,2]. Characterized by an acute disruption of cerebral blood flow—either ischemic or hemorrhagic—stroke frequently results in sensorimotor impairments that compromise mobility, balance, sometimes cognition communication swallowing, and overall independence [3]. Although advances in acute management have improved lifesaving, a substantial proportion of survivors experience chronic neurological and/or psycho-emotional affects that significantly impact areas including quality of life [4]. Among these, post-stroke spasticity is one of the most prevalent and functionally disabling complications [5].

Spasticity is traditionally defined as a velocity-dependent increase in tonic stretch reflexes, resulting from upper motor neuron lesions [6]. However, contemporary understanding recognizes post-stroke hypertonia as a complex phenomenon involving both neural and non-neural components. Neural mechanisms include hyperexcitability of the stretch reflex and altered descending inhibitory control. After stroke, damage to corticospinal and corticoreticulospinal pathways reduces inhibitory modulation of the brainstem and spinal cord, favoring increased excitability of reticulospinal projections and abnormal activation of alpha and gamma motor neurons. This imbalance contributes to stretch reflex hyperexcitability, exaggerated velocity-dependent muscle responses, and impaired reciprocal inhibition [7]. Non-neural mechanisms involve structural and biomechanical changes in muscle and connective tissue, such as increased passive stiffness, altered viscoelastic properties, shortened muscle fascicles, residual intermolecular liaisons between actin and myosin, and extracellular matrix remodeling [8,9,10]. Over time, these peripheral adaptations may become partially independent of central neural drive, contributing to contracture formation and persistent biomechanical constraints [11]. It should be noted that the pathophysiological pattern of neurogenic muscle hypertonia is complex and may initially be associated with the development of muscle shortening, altered viscoelastic properties, and progressive contracture formation. In the early stages after stroke, denervated or paretic muscles may remain for prolonged periods in shortened positions because of impaired voluntary activation, reduced range of motion, abnormal postural patterns, and insufficient antagonist muscle activity. This persistent shortening may promote structural adaptations within the muscle–tendon unit, including loss of sarcomeres in series, increased passive stiffness, changes in connective tissue composition, and collagen accumulation, making contracture one of the primary non-neural mechanisms contributing to increased resistance to passive movement. Subsequently, particularly in the post-acute and chronic stages, the loss of descending inhibitory and modulatory inputs from supraspinal motor centers leads to an unmasking of hyperactive spinal reflex activity [12]. As this spinal activity is no longer sufficiently counterbalanced by upper motor neuron control, especially through pyramidal and other descending inhibitory pathways, stretch reflex excitability increases, motor neuron thresholds may be reduced, and abnormal velocity-dependent muscle activation becomes more evident. Consequently, the clinical pattern of spasticity gradually emerges as the result of both neural mechanisms, such as reflex hyperexcitability, and non-neural peripheral changes, such as muscle stiffness, shortening, and contracture [13].

In the lower limbs—the focus of this paper, as anticipated in the title—spasticity frequently affects the plantar flexors, hamstrings, quadriceps, and hip adductors, leading to equinus or equinovarus foot positioning, reduced knee flexion during swing, decreased stride length, asymmetrical body loading, and impaired postural adjustments [14]. These alterations directly impact gait efficiency, dynamic balance, and overall stability [15]. Consequently, post-stroke spasticity is strongly associated with reduced walking speed, increased fall risk, and diminished participation in daily activities [16]. Addressing both neural overactivity and secondary biomechanical changes is therefore essential for comprehensive neurorehabilitation-related interventions [6].

BoNT-A has become a cornerstone in the focal management of post-stroke spasticity. Acting at the neuromuscular junction, BoNT-A inhibits acetylcholine release, producing a reversible reduction in muscle overactivity [17,18,19]. Clinically, this neuromodulatory intervention aims to reduce abnormal synergies, facilitate antagonist activation, improve passive range of motion, and create a therapeutic window for targeted rehabilitation with the purpose of restoring as completely as possible the complex, harmonized/ purposeful movements towards recovering as much as possible normal motor control. BoNT-A is particularly indicated when a limited number of muscles contribute disproportionately to functional impairment [20].

Despite widespread use, the translation of reduced muscle tone into meaningful functional gains—such as improved gait, balance, and mobility—remains variable [21]. Traditional clinical scales, including the Modified Tardieu Scale [22] and the Modified Ashworth Scale [23], primarily assess resistance to passive movement and are limited by ordinal scoring, examiner dependency, and inability to distinguish neural from mechanical contributions to hypertonia [24]. As a result, there is increasing recognition of the need for objective, quantitative, and non-invasive methods to monitor changes in muscle biomechanical and viscoelastic properties following BoNT-A, particularly after the first injection cycle, when early responsiveness may inform subsequent therapeutic planning [25,26].

Non-invasive biomechanical assessment tools have emerged as promising adjuncts in this context. Myotonometry, performed using devices such as the MyotonPRO digital palpation device (Myoton AS, Tallinn, Estonia) [27], enables quantification of mechanical and viscoelastic muscle properties through the application of a brief mechanical impulse and recording of tissue oscillation responses. Parameters commonly derived include oscillation frequency (reflecting intrinsic muscle tone), dynamic stiffness, logarithmic decrement (related to elasticity), relaxation time, and creep. These measurements provide insight into the passive mechanical behavior of muscle tissue and may help differentiate structural stiffness from reflex-mediated resistance. Importantly, myotonometric assessment is portable, reproducible, and suitable for repeated measurements across time points, making it particularly relevant for longitudinal monitoring after neuromodulation and/within rehabilitation endeavors [28,29,30,31].

Beyond muscle-level assessment, understanding the functional repercussions of biomechanical changes requires system-level evaluation of postural control and mobility. Instrumented stabilometric platforms, such as the ProKin 252 proprioceptive-stabilometric system (TecnoBody S.p.A., Dalmine, Italy) [32], allow objective analysis of balance performance through center-of-pressure (COP) metrics, sway velocity, sway area, directional control, and weight distribution symmetry [33]. These platforms can assess both static and dynamic conditions, offering insights into proprioceptive integration, anticipatory postural adjustments, and sensorimotor coordination. Given that lower-limb spasticity directly influences ankle and knee strategies for postural stabilization, stabilometric measures may be sensitive to functional changes following BoNT-A intervention [34,35,36].

The integration of localized biomechanical monitoring (e.g., Myoton [27]) with functional stabilometric assessment (e.g., ProKin [32]) provides a comprehensive framework to examine whether reductions in spastic muscle stiffness and altered viscoelastic properties translate into measurable improvements in balance control and functional mobility. This multidimensional approach aligns with contemporary rehabilitation paradigms emphasizing impairment-level, activity-level, and participation-level outcomes.

Although individual studies have investigated the effects of BoNT-A on spasticity and functional outcomes, the literature remains heterogeneous with respect to assessment tools, timing of evaluation, targeted muscle groups, and reported endpoints. Moreover, the specific relationship between early biomechanical changes in spastic musculature after the first course of BoNT-A and subsequent functional mobility and balance outcomes has not been systematically synthesized [24,26]. A focused examination of non-invasive monitoring strategies is particularly relevant, as these methods may offer objective biomarkers of treatment response and inform individualized rehabilitation planning (especially in the era of P4 Medicine) [37,38,39].

In accordance with the Preferred Reporting Items for PRISMA principles [40], the present systematic literature review aims to synthesize current evidence on the non-invasive monitoring of biomechanical and viscoelastic properties of spastic musculature in post-stroke patients, following the first course of BoNT-A neuromodulation, and to examine the reported repercussions on functional mobility and balance. By identifying methodological approaches, outcome measures, and reported associations between muscle-level changes and functional performance, this review seeks to clarify current evidence gaps and support future research directions in quantitative neurorehabilitation-related assessment [41,42].

Ultimately, establishing robust links between objective muscle-property measurements and functional balance outcomes may enhance clinical decision-making, optimize dosing and muscle selection strategies for BoNT-A therapy, and contribute to more precise, personalized rehabilitation pathways for individuals recovering from stroke. Because spasticity is a complex disabling condition, mainly by restraining both voluntary/active and passive motricity/mobility, and sometimes by causing pain, which further enhances mobility restraints, it consequently impedes, through this interplay, at least two functionally important basic abilities—standing and normal gait patterns, including balance impairment. Therefore, the combination of BoNT-A, which is the main spasmolytic treatment, its consequent favorable outcomes on mobility and balance, and the biomechanical viscoelastic muscle properties, representing the principal non-neurological/rheological component of muscle hypertonia and also being strongly connected to contracture, is clinically important. In our systematic literature review, we sought to identify how this meaningful clinical and functional issue is tackled in the available literature [43].

## 2. Materials and Methods 

### 2.1. Study Design and Research Question

In order to discover the current state of research and the level of knowledge regarding our topic, a systematic literature review was conducted. The selection and filtering of relevant studies were performed according to the PRISMA methodology [40] in order to ensure a transparent and structured process of identification, screening, eligibility assessment, and inclusion of scientific articles.

This systematic review addressed a specific research question structured according to the PICO framework [44]. The main aim was to investigate whether the effects of botulinum toxin neuromodulation in post-stroke hypertonia are evaluated not only through clinical outcomes but also through non-invasive assessment of spastic muscle properties, with particular attention to their relationship with mobility and balance. The PICO framework is described below:

- Population (P): Adult patients aged ≥18 years with post-stroke spasticity, regardless of stroke type, time since stroke onset, affected limb, or severity of motor impairment.
- Intervention (I): Botulinum toxin treatment used as a neuromodulatory intervention for post-stroke spasticity. Particular interest was given to studies that evaluated the effects of botulinum toxin through non-invasive assessment methods, including but not limited to clinical spasticity scales, ultrasound-based evaluation, elastography, myotonometry, biomechanical assessment, gait analysis, balance assessment, and stabilometric evaluation.
- Comparison (C): Baseline pre-treatment status, post-treatment follow-up moments, standardized clinical assessment instruments (using standardized instrumental tools such as both myotonometry and stabilometry), conventional rehabilitation, and BoNT-A administration combined with balance training based on advanced stabilometric devices or different control/comparator interventions are considered, depending on the design of each included study.
- Outcomes (O): Primary outcomes included changes in spasticity and spastic muscle properties, such as muscle tone, stiffness, elasticity, viscoelastic behavior, passive resistance, range of motion, and contracture-related features. Secondary outcomes included functional parameters such as mobility, gait performance, balance, postural control, motor recovery, functional independence, and quality of life when reported.

Therefore, the main research question of this systematic review is as follows: in adult post-stroke patients with spasticity (P), does botulinum toxin neuromodulation, assessed through non-invasive clinical, biomechanical, or functional methods (I), compared with baseline status, standard assessment, or alternative therapeutic approaches (C), provide measurable changes in spastic muscle properties and improve mobility and balance outcomes (O)?

### 2.2. Search Strategy

A systematic literature search was performed using combined controlled vocabulary and free-text search terms, applied contextually according to the syntax of each database. The complete keyword combinations are presented in Table 1. Relevant studies were identified through searches of international electronic databases such as ScienceDirect [45] (last accessed on 13 April 2026), PubMed [46] (last accessed on 13 April 2026), PubMed Central (PMC) [47] (last accessed on 13 April 2026), Physiotherapy Evidence Database (PEDro) [48] (last accessed on 13 April 2026), IEEE Xplore [49] (last accessed on 13 April 2026), and Web of Science [50] (last accessed on 13 April 2026). Web of Science was additionally used to verify the Institute of Scientific Information (ISI) [51] indexing status of the journals in which the selected articles were published. The search was restricted to studies published between 1 January 2023 and 31 December 2025.

### 2.3. Eligibility Criteria

Eligibility criteria included (1) peer-reviewed journal articles, (2) studies involving adult patients with spasticity following stroke, (3) administration of botulinum toxin as part of the therapeutic intervention, (4) availability of the full text free of charge and (5) publication in ISI-indexed journals, in the English language, and within the above-mentioned timed frame. Studies involving pediatric populations, non-stroke-related spasticity, conference abstracts, editorials, and were not published in ISI rated journals nor written in English were excluded. The study selection process, including identification, screening, eligibility assessment, and final inclusion, was performed following PRISMA methodological standards.

### 2.4. Study Quality Appraisal

In addition to the PRISMA-based selection process, the scientific impact and methodological quality of each paper were also considered. In order to quantify the quality of an article we took into consideration the impact that the article has, calculated using a customized PEDro-inspired approach, based on the weighted number of citations per year [52]. First, the number of citations per year (CPY) is computed for each article using Equation (1):

(1) $${CPY}_{i}=\frac{{TC}_{i}}{YC-{YP}_{i}}$$

where *CPY*_i_ stands for the citations per year of article *i*, *TC_i_* stands for the total number of citations for article *i*, *YP_i_* is the year of publication of article *i* and *YC* is the current year when the database interrogation was done.

Next, the average number of citations per year *ACPY* was calculated using Equation (2):

(2) $$ACPY=\frac{\sum_{i=1}^{N}{CPY}_{i}}{N}$$

where *ACPY* is the calculated value of the average number of citations per year and *N* is the number of articles.

The ACPY indicator is then considered to be corresponding to the PEDro score of 5 and each of the articles PEDro score is calculated using Equation (3).

(3) $${PEDro}_{i}=min\left(\left[\frac{5*\;{CPY}_{i}}{ACPY}\right],10\right)$$

The final calculation includes also a rounding to the nearest integer value and it limits the upper resulting value to 10; therefore, any article that has a PEDro score of more than 10 will be truncated to the max value of 10.

Methodological quality was further assessed using the PEDro scale, with studies scoring at least 4 points considered eligible for inclusion, corresponding to a minimum level of “fair quality” on the PEDro classification [53].

**Figure 1:**
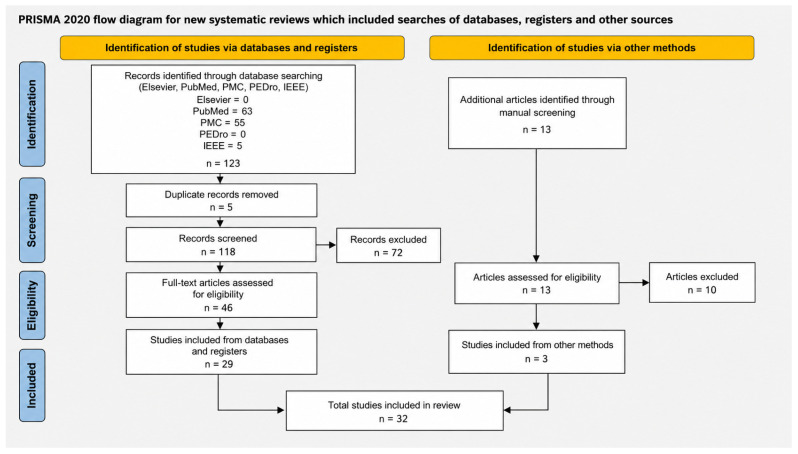
PRISMA-type diagram [55] specific to our systematic literature review.

Following all previous PRISMA screening and eligibility stages (and PRISMA checklist), as illustrated in Figure 1, the systematic search yielded a total of 123 records. After duplicate removal, 118 records remained and were screened based on title and abstract. Subsequently, 46 articles were selected for full-text assessment, while the 72 excluded articles were removed according to our customized qualitative filtering method based on citation impact and methodological quality, rather than because of language restrictions or lack of free full-text availability. After full-text review, 29 studies fulfilled all eligibility criteria and were included in the final synthesis.

The selection and evaluation of individual articles were conducted independently and concurrently by three authors. Any disagreements regarding study eligibility, methodological quality, or final inclusion were resolved through discussion using a Delphi-type consensus process [54].

To enhance and consolidate the related knowledge base, additional freely available bibliographic resources from the literature were also used within a balanced overall approach, mainly to support Section 1 and Section 4.

To ensure additional methodological rigor and transparency, this systematic literature review was registered in the International Prospective Register of Systematic Reviews (PROSPERO) database under the number CRD420261389647.

## 3. Results

The 29 articles included after applying the PRISMA selection method and the 3 articles included after the manual searching, in this systematic literature review, are presented in Table 2 and Table 3. In order to frame within a reasonable length of this paper we have chosen the below presented tables as the form of presenting the results of our quest. The tables provide a brief description of each study, highlighting its main focus in relation to botulinum toxin neuromodulation, post-stroke spasticity, muscle properties, non-invasive assessment methods, and associated functional outcomes such as mobility and balance.

## 4. Discussion

As observed in Section 3, the 32 articles included in this systematic review are relevant to the broader topic of botulinum toxin neuromodulation in post-stroke spasticity. However, the available evidence does not fully address the specific focus of the present review, namely the non-invasive assessment of spastic muscle biomechanical and viscoelastic properties after botulinum toxin treatment, and the relationship between these changes and functional outcomes related to mobility, gait, postural control, and balance. Although the included studies confirm the clinical relevance of BoNT-A in the management of post-stroke spasticity, only a limited number specifically explore its effects in connection with objective muscle-property assessment, gait analysis, balance evaluation, or functional mobility.

An important aspect that should be acknowledged is the heterogeneity of the included literature. The analyzed studies differ substantially in terms of design, population characteristics, stroke chronicity, injected muscles, BoNT-A dosage and formulation, guidance technique, rehabilitation protocol, follow-up duration, and outcome measures. This heterogeneity limits the possibility of drawing uniform conclusions regarding the direct relationship between BoNT-A-induced muscle changes and functional recovery. Nevertheless, because studies directly addressing the full combination of BoNT-A administration, non-invasive muscle-property assessment, and balance or mobility outcomes remain scarce, it was necessary to include and discuss a broader range of related studies. Therefore, the present discussion integrates all 32 articles, not because they are methodologically or thematically identical, but because together they provide the closest available evidence surrounding the research gap addressed by this review.

A large proportion of the included literature focuses mainly on the clinical use of botulinum toxin in post-stroke spasticity. These studies discuss aspects such as treatment timing [57,59,69,79], injection guidance [58,67,81], muscle selection, treatment goals [43,60], long-term management [20,68,80], and clinical effectiveness. This confirms the importance of botulinum toxin in the rehabilitation of post-stroke patients with spasticity. Nevertheless, in many of these studies, treatment effects are evaluated primarily through clinical scales such as the Modified Ashworth Scale, Modified Tardieu Scale, passive range of motion (ROM), pain scores, or goal-attainment measures [56,64,65,69,76,77]. As mentioned previously, although these evaluation tools are useful in clinical practice, they provide limited information about the underlying biomechanical and viscoelastic changes occurring in spastic muscles after treatment.

Some of the included articles are more closely related to mobility and gait outcomes. For instance, studies investigating botulinum toxin injection in lower-limb muscles, such as the rectus femoris in patients with stiff-knee gait, suggest that reducing focal spasticity may contribute to improvements in walking performance and gait mechanics [66]. Other studies examine the combination of botulinum toxin with rehabilitation strategies, such as robotic gait training or structured physical therapy, showing that post-injection rehabilitation may enhance functional outcomes [68,70,74,82]. The randomized controlled trial by Yu et al. further strengthens this direction, as it specifically evaluated gait and postural control after BoNT-A injection for lower-limb spasticity after stroke, making it particularly relevant to the mobility and balance component of the present review [35]. However, balance outcomes are less consistently assessed, and only a small number of studies directly connect botulinum toxin treatment with postural control, stabilometric parameters, or balance performance.

Another important finding is that some studies address muscle properties, but this is often done indirectly. Thus, several articles discuss muscle stiffness, contracture risk, passive range of motion, pain, muscle shortening, or spastic hypertonia (see our related comments in Section 1), all of which being related to the mechanical behavior of the neuropathological hypertonic muscles [57,59,69,77,79]. However, few studies use objective non-invasive technologies, such as ultrasound-based methods, elastography, myotonometry, or other biomechanical assessment instruments, to quantify changes in muscle elasticity, stiffness, or viscoelastic properties after botulinum toxin treatment. Therefore, the current literature provides only partial evidence regarding how botulinum toxin modifies the physical properties of post-stroke spastic muscles. In this regard, the exploratory study by Simic et al. is particularly relevant, as it directly investigated the short-term effects of BoNT-A on muscle stiffness in stroke patients using shear-wave elastography [83]. This study supports the feasibility and clinical value of objective, non-invasive muscle-property monitoring after BoNT-A treatment. However, such evidence remains limited, and elastography-based evaluation is still rarely integrated with functional balance or mobility outcomes. Similarly, although ultrasound-guided injection studies support the use of non-invasive imaging for muscle identification and injection precision [58,67,81], these approaches are mainly used for treatment guidance rather than for follow-up quantification of biomechanical or viscoelastic muscle changes.

The additional study by Picelli et al. also contributes to the discussion by emphasizing the possible importance of early BoNT-A administration in post-stroke spasticity and its potential relationship with motor recovery [84]. This is relevant because early modulation of spasticity may theoretically reduce secondary complications such as muscle shortening, pain, contracture, and maladaptive motor patterns. However, while early treatment may improve the clinical trajectory of recovery, further studies are needed to determine whether early BoNT-A-induced reductions in spasticity are accompanied by measurable changes in muscle stiffness, elasticity, postural control, and functional mobility.

Overall, the included studies show that BoNT-A is widely investigated and clinically relevant in post-stroke spasticity management. Some articles focus on treatment timing, injection technique, target-muscle selection, treatment goals, and long-term management; others address rehabilitation combinations, gait, mobility, pain, passive range of motion, or stiffness-related consequences. The three additional studies found within the manual searching of the literature further support the relevance of early BoNT-A treatment, objective measurements such as stiffness assessment using elastography, and lower-limb functional outcomes related to gait and postural control. However, these dimensions are rarely studied together within the same research protocol. This represents the main gap identified by the present review: the insufficient integration of BoNT-A neuromodulation, non-invasive assessment of muscle mechanical and viscoelastic properties, and objective evaluation of mobility and balance outcomes.

Therefore, the present review highlights the need for future controlled longitudinal studies combining clinical, biomechanical, and functional assessment methods. Future research should not only determine whether BoNT-A reduces spasticity, but also clarify how changes in muscle stiffness, elasticity, tone, and viscoelastic behavior influence gait, balance, and functional mobility in post-stroke patients with muscle hypertonia. Such studies should ideally include baseline pre-treatment assessment, clearly defined therapeutic groups, standardized BoNT-A administration, structured rehabilitation protocols, and repeated post-treatment follow-up evaluations. Instrumental tools such as myotonometry, elastography, gait analysis, and stabilometric platforms should be used together with standardized clinical–functional scales in order to obtain a more complete understanding of treatment response.

The scarcity of studies directly matching the full focus of this review justifies the inclusion of a heterogeneous but clinically relevant body of literature. The current evidence supports the plausibility that BoNT-A treatment, especially when combined with rehabilitation, may improve spasticity-related impairment and functional outcomes after stroke. However, the relationship between local muscle-property changes and global improvements in gait, balance, and mobility remains insufficiently clarified. This finding strengthens the rationale for future integrative research protocols combining BoNT-A treatment, non-invasive muscle-property measurements such as myotonometry and instrumented balance and mobility assessment.

## 5. Limitations

The present review has several limitations that should be acknowledged. First, despite the continuous interest in the management of post-stroke spasticity, only a limited number of recent studies specifically addressed an integrated approach combining BoNT-A administration, objective assessment of hypertonic muscle-property changes, and functional outcomes related to postural control, balance, and gait. This reduced the possibility of drawing strong conclusions regarding the direct relationship between neuromodulation-induced changes in muscle properties and clinically meaningful functional improvements. Second, the relatively narrow time frame of the search may have excluded earlier studies that could provide additional background or complementary evidence regarding BoNT-A treatment, objective muscle-property assessment, mobility, and balance outcomes in post-stroke spasticity. Third, the restriction to English-language, free full-text, and ISI-indexed articles may have limited the breadth of the available evidence and may have introduced a degree of selection bias, although these criteria were applied in order to ensure accessibility, reproducibility, and a minimum level of journal-quality filtering. Fourth, the included studies were characterized by considerable clinical and methodological heterogeneity, particularly regarding patient characteristics, stroke chronicity, muscles targeted for BoNT-A injection, rehabilitation protocols, follow-up periods, and outcome measures. Fifth, no formal risk-of-bias assessment was performed, mainly because of the marked heterogeneity of the included studies in terms of design and methodological structure, which included randomized controlled trials, observational studies, reviews, technical papers, surveys, epidemiological analyses, and health-economic studies. In addition, because only a very limited number of studies could be considered potentially suitable for quantitative comparison and these differed substantially in target muscle groups, intervention protocols, comparators, and functional outcomes, no meta-analysis was performed. Therefore, the present review provides a structured qualitative synthesis rather than a quantitative estimate of treatment effect. Furthermore, not all studies used instrumental assessment methods such as myotonometry, elastography, stabilometry, or gait analysis, which limited the comparability of objective biomechanical and functional findings. In addition, differences in BoNT-A dosage, injection technique, guidance method, and concomitant rehabilitation interventions may have influenced the reported outcomes. Finally, because many available studies focused mainly on clinical spasticity scales rather than on combined biomechanical and balance-related assessments, the evidence remains insufficient to fully clarify how changes in hypertonic muscle properties after BoNT-A treatment translate into improvements in mobility and balance. These limitations reinforce the need for future prospective studies with standardized protocols, homogeneous outcome measures, formal risk-of-bias assessment, and integrated evaluation of BoNT-A effects on muscle viscoelastic properties, mobility, and balance.

## 6. Conclusions

This systematic literature review aimed to identify and synthesize recent evidence regarding the non-invasive monitoring of biomechanical and viscoelastic properties of spastic musculature in post-stroke patients following botulinum toxin administration, with particular attention to possible repercussions on functional mobility, postural control, and balance. The available recent literature confirms the continued scientific and clinical interest in BoNT-A treatment for post-stroke spasticity, especially in relation to muscle-tone reduction and functional recovery. However, the present review also highlights that the existing evidence remains fragmented, as most studies approach these aspects separately rather than through a unified, multidimensional assessment protocol.

To our knowledge, the recent literature does not provide sufficient studies that simultaneously integrate all the components central to this research direction: post-stroke spasticity, BoNT-A administration, objective myotonometric assessment of muscle viscoelastic properties, and proprioceptive–stabilometric evaluation, such as that performed using advanced devices like ProKin. This absence of fully integrated investigations does not diminish the relevance of the topic; on the contrary, it emphasizes an important research gap and supports the need for future studies capable of correlating local muscle-property changes with global functional outcomes. The current evidence mainly shows that there is still a lack of studies combining BoNT-A treatment, objective muscle-property assessment, and mobility or balance outcomes.

Based on these findings, future research should move beyond isolated clinical spasticity assessment and should incorporate combined evaluation models. More specifically, longitudinal controlled studies are recommended, including baseline pre-treatment assessment, clearly defined therapeutic groups, standardized BoNT-A administration protocols, conventional rehabilitation or balance-oriented rehabilitation comparators, and repeated post-treatment follow-up evaluations. Such studies should include objective monitoring of muscle properties through tools such as myotonometry, functional balance and postural-control assessment using stabilometric platforms, and standardized clinical–functional outcome measures related to mobility, gait, and daily functioning.

In this context, a future research protocol may be structured around pre-treatment clinical and instrumental evaluation, followed by comparison between different therapeutic approaches, such as BoNT-A administration combined with conventional rehabilitation versus BoNT-A administration associated with targeted balance training using an advanced stabilometric device. Repeated monitoring at predefined follow-up moments would allow the evaluation of changes in muscle stiffness, tone, elasticity, postural control, gait parameters, and functional mobility. Such an approach could help clarify whether objective modifications in hypertonic muscle properties after BoNT-A treatment are directly associated with clinically meaningful improvements in balance and mobility.

Therefore, the present review supports the need for more integrative, clinically oriented, and instrumentally monitored research in post-stroke spasticity rehabilitation. Combining focal neuromodulation, quantitative assessment of muscle biomechanical properties, and advanced balance evaluation may contribute to more precise treatment monitoring, individualized rehabilitation planning, and improved functional outcomes in post-stroke patients.

## Figures and Tables

**Table 1:** Combination of keywords/syntaxes, contextually, searched within our PRISMA quest.

Keywords	ScienceDirect	PubMed	PMC	PEDro	IEEE	Total
“Botulinum toxin” AND “Post Stroke” AND “Neuromodulation” AND “Muscle Spasticity”	0	63	50	0	3	116
“Botulinum toxin” AND “Post Stroke” AND “Neuromodulation” AND “Muscle elasticity estimation” AND “Viscoelastic properties”	0	0	0	0	0	0
“Botulinum toxin” AND “Post Stroke” AND “Neuromodulation” AND “Muscle elasticity estimation” AND “Biomechanical properties”	0	0	0	0	0	0
“Botulinum toxin” AND “Post Stroke” AND “Neuromodulation” AND “Muscle elasticity estimation” AND “Non-invasive monitoring”	0	0	0	0	0	0
“Botulinum toxin” AND “Post Stroke” AND “Neuromodulation” AND “Neuro-rehabilitation” AND “Motor recovery”	0	0	4	0	0	4
“Botulinum toxin” AND “Post Stroke” AND “Neuromodulation” AND “Muscle Spasticity” AND “Balance Impairment”	0	0	1	0	2	3
“Botulinum toxin” AND “Post Stroke” AND “Neuromodulation” AND “Neuro-rehabilitation” AND “Proprioceptive-Stabilometric Assessment”	0	0	0	0	0	0
**Total**	**0**	**63**	**55**	**0**	**5**	**123**

**Table 2:** Brief description of the 29 studies included in our systematic literature review by database searching.

No.	Study/Design	Sample & Follow-Up	Intervention & Target Muscles	Outcome Measures	Main Findings/Relevance	Score
1	**Effectiveness of Extracorporeal Shock Wave Therapy after Botulinum Toxin Injection for Post-Stroke Upper-Extremity Spasticity: A Randomized Controlled Study** *Lee J.; Yang S.N.* **Design/type:** Randomized controlled adjunctive-treatment study.	**Sample:** 16 patients with post-stroke upper-extremity spasticity. **Follow-up:** Baseline, 3 weeks and 3 months after injection.	**Intervention:** BoNT-A injection plus focused extracorporeal shock wave therapy (ESWT) versus BoNT-A injection alone. **Target:** Individualized upper-limb flexor pattern; injected muscles included biceps brachii, brachioradialis, pronator teres, FCR, FCU, FDP and FDS; ESWT was applied to biceps brachii or FCR.	MAS, MTS, UE-FMA, MBI and ARAT.	Both groups improved clinically in elbow and wrist flexor spasticity after BoNT-A. The ESWT adjunct showed additional finger-flexor spasticity and MTS-R1 changes at follow-up, but between-group functional gains were limited and UE-FMA, MBI and ARAT did not show significant functional improvement. This study is useful for adjunctive upper-limb spasticity management, but it does not assess gait, balance or objective muscle mechanical properties [56].	**10**
2	**The effectiveness of early interventions for post-stroke spasticity: a systematic review** *van Tilborg N.A.W.; de Groot V.; Meskers C.G.M.* **Design/type:** Systematic review; secondary evidence, not a primary clinical trial.	**Sample:** NA for individual patient sample in this table; includes multiple early-intervention studies. **Follow-up:** Variable across included studies.	**Intervention:** Early interventions for post-stroke spasticity, including BoNT-A and rehabilitation-based approaches. **Target:** Upper- and/or lower-limb muscles depending on included studies.	Mainly clinical spasticity and functional outcomes across included studies; exact measures vary by study.	The review supports the clinical importance of early identification and treatment of post-stroke spasticity, including BoNT-A where appropriate. However, because it is secondary evidence, it should be used as background only. It does not directly provide a single extractable BoNT-A protocol with target muscles, follow-up and objective muscle-property outcomes [57].	**10**
3	**The Elias University Hospital Approach: A Visual Guide to Ultrasound-Guided Botulinum Toxin Injection in Spasticity. Part I—Distal Upper Limb Muscles** *Popescu M.N.; Capet C.; Beiu C.; Berteanu M.* **Design/type:** Technical/visual guide; contextual injection-method paper.	**Sample:** NA. **Follow-up:** NA.	**Intervention:** Ultrasound-guided BoNT injection technique. **Target:** Distal upper-limb muscles involved in wrist, finger and hand spasticity.	NA; procedural anatomical guidance rather than clinical outcome measurement.	This article is relevant for safe and precise ultrasound-guided targeting of distal upper-limb muscles. It should be cited as procedural background, not as evidence of functional improvement, because it does not report mobility, balance, follow-up outcomes or objective muscle-property assessment after BoNT-A [58].	**10**
4	**Early versus late injections of Botulinumtoxin type A in post-stroke spastic movement disorder: A literature review** *Wissel J.; Ri S.; Kivi A.* **Design/type:** Literature review; secondary evidence.	**Sample:** NA for a single patient cohort. **Follow-up:** Variable across cited studies.	**Intervention:** Early versus delayed BoNT-A injection strategies in post-stroke spastic movement disorder. **Target:** Post-stroke spastic movement patterns; target muscles vary by clinical presentation.	Clinical spasticity, disability and complication-related outcomes discussed across the literature.	The review supports the rationale that earlier BoNT-A treatment may reduce long-term spasticity-related disability, pain and soft-tissue complications. It is useful for Section 4 but should not be treated as a primary study in the synthesis table because it does not provide original patient-level outcome data [59].	**10**
5	**Best Practice Guidelines for the Management of Patients with Post-Stroke Spasticity: A Modified Scoping Review** *Suputtitada A.; Chatromyen S.; Chen C.P.C.; Simpson D.M.* **Design/type:** Modified scoping review/guideline-oriented review.	**Sample:** NA for a primary patient cohort. **Follow-up:** Variable/NA.	**Intervention:** Multimodal management of post-stroke spasticity, including BoNT-A, rehabilitation and assessment strategies. **Target:** Upper- and lower-limb spasticity depending on treatment goals.	Guideline-level clinical assessment and management domains; outcome measures vary by evidence source.	This paper provides broad best-practice context for BoNT-A within multidisciplinary post-stroke spasticity care. It supports individualized treatment goals, appropriate assessment and adjunctive rehabilitation, but it is not a primary trial and therefore should not be pooled with intervention studies [60].	**10**
6	**Multimodal therapy and use of adjunctive therapies to BoNT-A in spasticity management: defining terminology to help enhance spasticity treatment** *Reebye R.; Jacinto L.J.; Balbert A.; Biering-Sorensen B.; Carda S.; Draulans N.; Molteni F.; O’Dell M.W.; Picelli A.; Santamato A.; Verduzco-Gutierrez M.; Walker H.; Wissel J.; Francisco G.E.* **Design/type:** Terminology/consensus-style review.	**Sample:** NA. **Follow-up:** NA.	**Intervention:** Definitions and framework for multimodal and adjunctive therapies used with BoNT-A. **Target:** Variable; depends on the spasticity pattern treated clinically.	NA as a direct clinical outcome study; discusses how outcome domains should be linked to adjunctive-treatment goals.	The article is useful for clarifying terminology such as adjunctive, combined and multimodal treatment after BoNT-A. It supports explaining why BoNT-A should be integrated with rehabilitation, stretching, robotics or other therapies, but it should remain contextual because it does not report a primary intervention cohort [61].	**10**
7	**Botulinum Neurotoxins: History, Mechanism, and Applications. A Narrative Review** *Monash A.; Tam J.; Rosen O.; Soreq H.* **Design/type:** Narrative review; mechanistic background.	**Sample:** NA. **Follow-up:** NA.	**Intervention:** Mechanisms and clinical applications of botulinum neurotoxins. **Target:** NA; not specific to a post-stroke target-muscle protocol.	NA for clinical outcomes in this table.	This review may be used to support the biological rationale of BoNT neuromodulation, including chemodenervation and reduction in excessive neuromuscular activity. It does not provide primary post-stroke data on muscle stiffness, gait, balance or follow-up response [62].	**10**
8	**Spasticity Treatment Beyond Botulinum Toxins** *Li S.; Winston P.; Mas M.F.* **Design/type:** Review article; contextual treatment overview.	**Sample:** NA. **Follow-up:** NA.	**Intervention:** Non-BoNT and complementary approaches for spasticity management. **Target:** Variable; not a BoNT-A target-muscle protocol.	NA for a primary BoNT-A outcome dataset.	The article places BoNT-A within a broader spasticity-management framework and is useful for discussing alternatives and adjuncts. It should not be considered a primary BoNT-A study because it does not provide a patient cohort with extractable intervention, target muscle and follow-up data [63].	**9**
9	**Long-Term Enhancement of Botulinum Toxin Injections for Post-Stroke Spasticity by Use of Stretching Exercises—A Randomized Controlled Trial** *Hwang I.S.; Ryu J.W.; Jin S.; Kim S.A.; Kim M.S.* **Design/type:** Prospective randomized double-blinded controlled trial.	**Sample:** 43 subacute stroke patients with upper-limb spasticity in the final analysis: 21 in the assisted-stretching group and 22 in the self-stretching control group. **Follow-up:** 6 months after injection.	**Intervention:** BoNT/A injection plus therapist-assisted structured stretching versus BoNT/A injection plus self-stretching instructions; both groups continued conventional rehabilitation. **Target:** Upper-limb flexor pattern; injected muscles included biceps brachii, brachioradialis, FCR, FCU, FDS and FDP. Shoulder stretching was included although pectoralis major was not injected.	Primary: MAS. Secondary: shoulder-pain VAS, K-MBI, FMA-UE, EQ-5D and EMG/RMS activity.	Structured stretching after BoNT/A prolonged the anti-spastic effect through 6 months compared with self-directed stretching and was associated with improvements in shoulder pain, activities of daily living and quality of life. A superiority effect for upper-limb motor recovery on FMA-UE was not evident. The study is relevant to adjunctive rehabilitation after BoNT-A but does not address gait, balance or instrumental muscle-property measurement [64].	**9**
10	**Combined effects and timing of robotic training and botulinum toxin on upper limb spasticity and motor function: a single-blinded randomized controlled pilot study** *Shin J.H.; Park G.; Kim H.; Cho D.Y.; Kwon S.* **Design/type:** Single-blinded randomized controlled pilot timing study.	**Sample:** 42 participants with chronic stroke-induced upper-limb spasticity were enrolled; 40 completed the study. **Follow-up:** Week 0, week 4 and week 8.	**Intervention:** Robotic training and BoNT injection were combined in different timing sequences across four groups, with BoNT and/or robotic training initiated at week 0 or week 4. **Target:** Upper-limb spasticity; elbow flexor and extensor spasticity were specifically followed, with individualized injected muscles according to clinical pattern.	Primary: FMA. Secondary: MAS of elbow flexors/extensors and robotic kinematic variables, including spectral arc length, mean speed, hand-path ratio and movement deviation.	Combined BoNT and robotic training produced better spasticity and motor-function effects than single interventions or no intervention. Immediate combined treatment produced the strongest early reduction in elbow MAS and kinematic improvement, whereas robotic training initiated one month after BoNT produced the strongest week-8 functional and kinematic gains. This supports careful timing of adjunctive rehabilitation but remains an upper-limb study without gait or balance outcomes [65].	**9**
11	**Selecting Goals and Target Muscles for Botulinum Toxin A Injection Using the Goal Oriented Facilitated Approach to Spasticity Treatment (GO-FAST) Tool** *Jacinto J.; Balbert A.; Bensmail D.; Carda S.; Draulans N.; Deltombe T.; Ketchum N.; Molteni F.; Reebye R.* **Design/type:** Clinical framework/goal-oriented tool paper.	**Sample:** NA for a primary intervention cohort in this table. **Follow-up:** NA.	**Intervention:** GO-FAST tool to connect patient-centered goals with BoNT-A target-muscle selection. **Target:** Variable; determined by goal, limb segment and spastic pattern.	Goal-oriented clinical assessment; supports selection of appropriate functional outcomes, but does not report a single trial outcome set.	The paper is useful for strengthening the Methods and Discussion sections by showing that target-muscle selection should be explicitly linked to functional goals. It does not provide primary outcome data, but it can justify a structured protocol using goal attainment, mobility or balance measures after BoNT-A [43].	**8**
12	**The Effect of Botulinum Toxin Type A Injection in the Rectus Femoris in Stroke Patients Walking With a Stiff Knee Gait: A Randomized Controlled Trial** *Tenniglo M.J.B.; Nene A.V.; Rietman J.S.; Buurke J.H.; Prinsen E.C.* **Design/type:** Triple-blind randomized placebo-controlled cross-over trial.	**Sample:** 26 adult stroke patients with stiff-knee gait. **Follow-up:** Post-injection assessment at approximately 4–6 weeks; 5-month washout between cross-over periods.	**Intervention:** 200 U BoNT-A injected into rectus femoris versus saline placebo, with cross-over after washout. **Target:** Rectus femoris.	Instrumented gait/kinematics with peak knee flexion and knee range of motion; functional walking outcomes including 6 MWT, walking speed/10 MWT-related measures, energy cost and perceived gait quality.	Rectus femoris BoNT-A improved sagittal knee kinematics during swing, with increased peak knee flexion and knee range of motion. A modest improvement in 6 MWT distance was reported, whereas hip kinematics did not significantly change. This is one of the most directly relevant lower-limb BoNT-A studies for mobility outcomes, although it did not include myotonometry, elastography or stabilometric balance assessment [66].	**8**
13	**A Bayesian Network Meta-Analysis and Systematic Review of Guidance Techniques in Botulinum Toxin Injections and Their Hierarchy in the Treatment of Limb Spasticity** *Asimakidou E.; Sidiropoulos C.* **Design/type:** Systematic review and Bayesian network meta-analysis; secondary evidence.	**Sample:** NA for a single clinical cohort; includes multiple guidance-technique studies. **Follow-up:** Variable across included studies.	**Intervention:** Comparison of guided versus non-guided BoNT injection approaches, including ultrasound, EMG and electrical stimulation guidance. **Target:** Upper- and/or lower-limb muscles depending on included trials.	Spasticity and clinical efficacy outcomes across included trials; measures vary.	The review supports the importance of accurate injection guidance for improving BoNT treatment precision. It is methodologically relevant to injection planning, but it should be cited as secondary evidence rather than as a primary study of mobility, balance or muscle mechanical response [67].	**8**
14	**Post-stroke spasticity: follow-up and functional implications of chronic long-term treatment with botulinum toxin** *Battaglia M.; Borg M.B.; Loro A.; Cosenza L.; Scotti L.; Picelli A.; Filippetti M.; Bertoni M.; Spina S.; Santamato A.; Carda S.; Baricich A.* **Design/type:** Long-term observational follow-up study.	**Sample:** Chronic post-stroke spasticity cohort; exact sample size should be verified from the full text before final submission. **Follow-up:** Long-term/chronic follow-up; exact duration should be verified from the full text.	**Intervention:** Repeated BoNT treatment in routine long-term management; no randomized comparator in the extracted table. **Target:** Individualized upper- and/or lower-limb target muscles depending on spastic pattern and goals.	Functional implications of long-term follow-up; exact scales should be verified from the full text.	This study is relevant because it addresses repeated BoNT use over time and its functional implications in chronic post-stroke spasticity. It can support discussion of maintenance treatment and follow-up planning, but exact sample size, outcome measures and follow-up duration should be checked directly in the full article before finalizing the manuscript table [68].	**7**
15	**Botulinum Toxin in Pain-Related Post-Stroke Limb Spasticity: A Meta-Analysis of Early and Late Injections** *Tamayo F.M.; Rosales R.; Wissel J.; Biering-Sorensen B.; Ellano J.N.; Simpson D.* **Design/type:** Meta-analysis; secondary evidence.	**Sample:** NA for a single patient cohort. **Follow-up:** Variable across included studies.	**Intervention:** BoNT-A for pain-related post-stroke limb spasticity, including comparisons of early and late injections across included studies. **Target:** Upper- and/or lower-limb muscles depending on pain-related spasticity pattern.	Pain, spasticity and related functional or range-of-motion outcomes across included studies.	The article supports the concept that pain relief is an important outcome after BoNT-A and may indirectly improve passive range of motion, positioning and participation in rehabilitation. It should be used as contextual evidence rather than as a primary study in the structured synthesis [69].	**7**
16	**Robotic gait training and botulinum toxin injection improve gait in the chronic post-stroke phase: A randomized controlled trial** *Cotinat M.; Celerier M.; Arquilliere C.; Flipo M.; Prieur-Blanc N.; Viton J.M.; Bensoussan L.* **Design/type:** Randomized controlled sequencing trial of rehabilitation after BoNT-A.	**Sample:** 33 chronic stroke participants with triceps surae spasticity causing gait impairment: Group A *n* = 15 and Group B *n* = 18. **Follow-up:** Two consecutive 2-week rehabilitation blocks after injection, with pre-test and post-test assessments across the sequence.	**Intervention:** BTx-A injection followed by Lokomat robotic gait training and conventional physiotherapy in different 2-week sequences. **Target:** Spastic triceps surae.	Primary: 6 MWT. Additional walking and balance-related measures reported in the study include 10 mWT, BBS and TUG.	Robotic gait training performed after BoNT-A improved walking performance more than conventional physiotherapy alone in chronic post-stroke gait impairment. This is highly relevant to mobility outcomes after lower-limb BoNT-A, especially because it combines toxin treatment with gait-oriented rehabilitation, but it still does not include objective muscle-property testing such as myotonometry or elastography [70].	**7**
17	**Canadian Physicians’ Use of Intramuscular Botulinum Toxin Injections for Shoulder Spasticity: A National Cross-Sectional Survey** *Kassam F.; Lim B.; Afroz S.; Boissonnault E.; Reebye R.; Finlayson H.; Winston P.* **Design/type:** National cross-sectional physician survey.	**Sample:** 50 Canadian physical medicine and rehabilitation physicians completed the survey. **Follow-up:** NA; cross-sectional survey.	**Intervention:** Survey of intramuscular BoNT injection practice for shoulder spasticity; no direct intervention cohort. **Target:** Shoulder muscles involved in post-stroke or acquired spasticity; specific muscle choices reflect physician practice patterns.	Survey responses on clinical practice, target selection and injection behavior.	This study is useful for describing real-world variation in shoulder-spasticity BoNT practice. It does not test efficacy or provide patient-level follow-up data, so it should be positioned as practice-pattern evidence rather than primary outcome evidence [71].	**7**
18	**Long-Term Management of Post-Stroke Spasticity with Botulinum Toxin: A Retrospective Study** *Falcone N.; Leo F.; Chisari C.; Dalise S.* **Design/type:** Retrospective long-term chart review.	**Sample:** 95 chronic stroke patients treated with BoNT-A. **Follow-up:** Long-term follow-up ranging approximately from 2 to 14 years.	**Intervention:** Repeated BoNT-A treatment over multiple cycles in routine management; no randomized comparator. **Target:** Individualized upper- and/or lower-limb muscles according to spasticity pattern and clinical goals.	Treatment duration, dose variability, injection intervals and dropout rates; clinical outcomes reported in the study context.	Repeated BoNT-A treatment was analyzed as a long-term management strategy. Injection intervals tended to extend over time and dosage showed an increasing trend, while discontinuation was most frequent in the earlier years of treatment. The study is valuable for treatment-continuity and service-planning discussion, but less directly informative for short-term mobility, balance or objective muscle mechanical response [20].	**6**
19	**A modified scoping review of interventions for global post stroke spasticity** *Suputtitada A.; Chatromyen S.; Chen C.P.C.; Simpson D.M.* **Design/type:** Modified scoping review; secondary evidence.	**Sample:** NA for a primary patient cohort. **Follow-up:** Variable/NA.	**Intervention:** Global post-stroke spasticity interventions, including BoNT-A, rehabilitation and other modalities. **Target:** Global/multi-pattern spasticity; upper and lower limb involvement depending on intervention.	Assessment and management domains across the included literature; exact outcomes vary.	This review provides broad context for multimodal treatment of post-stroke spasticity but does not provide a single primary clinical dataset. It should be used to support background statements and not mixed with primary BoNT-A efficacy studies in a quantitative synthesis [72].	**6**
20	**The Incidence and Outpatient Medical Care of Patients with Post-Stroke Spasticity** *Rakers F.; Weise D.; Hamzei F.; Musleh R.; Schwab M.; Jacob J.; Alibone M.; Gunther A.* **Design/type:** Epidemiological/health-services analysis.	**Sample:** Large administrative or insurance-based population of stroke patients; exact denominator should be verified from the full text. **Follow-up:** Administrative observation period; not a post-injection follow-up study.	**Intervention:** No therapeutic intervention; describes incidence and outpatient care patterns. **Target:** NA; not a target-muscle treatment study.	Incidence of post-stroke spasticity and outpatient medical-care utilization.	This study is useful for framing the burden of post-stroke spasticity and outpatient care needs. It does not evaluate BoNT-A effects, target muscles, gait, balance or muscle properties, so it should be used only as epidemiological context [73].	**6**
21	**Treatment of adult spasticity with Botox (onabotulinumtoxinA): Development, insights, and impact** *Esquenazi A.; Jost W.H.; Turkel C.C.; Wein T.; Dimitrova R.* **Design/type:** Review/development and clinical-insight article.	**Sample:** NA for a single patient cohort. **Follow-up:** Variable across cited studies.	**Intervention:** OnabotulinumtoxinA use in adult spasticity, including post-stroke applications. **Target:** Upper- and lower-limb muscles depending on approved indication and clinical pattern.	Clinical efficacy, safety and functional considerations summarized from prior evidence.	The article provides background on Botox development, dosing concepts and adult spasticity indications. It is useful for contextualizing BoNT-A as an established treatment, but it is not a primary post-stroke trial with extractable follow-up outcomes [74].	**6**
22	**Botulinum Toxin Utilization, Treatment Patterns, and Healthcare Costs Among Patients with Spasticity or Cervical Dystonia in the US** *Hull M.; Anupindi V.R.; DeKoven M.; He J.; Bouchard J.* **Design/type:** Retrospective claims database/health-economic study.	**Sample:** US patients with spasticity or cervical dystonia; exact post-stroke subgroup size should be verified from the full text. **Follow-up:** Two-year observation period in the claims analysis.	**Intervention:** Analysis of BoNT utilization, treatment patterns and costs; no direct clinical intervention protocol. **Target:** NA for target-muscle extraction; based on claims data rather than injection-level anatomy.	Healthcare utilization, treatment patterns, costs and time to treatment identification.	This study provides real-world context on underuse, timing and cost burden of BoNT-related care. It does not provide clinical outcome measures such as MAS, gait tests, balance scores or muscle mechanical properties, so it should be retained as health-services background rather than primary efficacy evidence [75].	**5**
23	**The Complex Role of Botulinum Toxin in Enhancing Goal Achievement for Post-Stroke Patients** *Sandulescu M.I.; Cinteza D.; Poenaru D.; Potcovaru C.G.; Paunescu H.; Coman O.A.* **Design/type:** Observational non-randomized clinical study.	**Sample:** 52 stroke survivors with upper-limb hypertonia treated in a rehabilitation setting. **Follow-up:** Short-term post-treatment assessment, approximately 20 +/− 5 days after baseline/treatment in the reported protocol.	**Intervention:** AbobotulinumtoxinA injection combined with rehabilitation; no randomized comparator. **Target:** Upper-limb spasticity pattern; frequently injected muscles included biceps brachii, FDS, pronator teres, brachialis and FDP, with less frequent FCU and FPL injection. Pectoralis major and proximal upper-limb muscles were also clinically relevant.	Goal-Attainment Scaling T-score (GAS-T), FIM, MRC motor-control measures, pain, spasticity and joint mobility measures.	Goal achievement after BoNT-A was influenced by prior BoNT exposure and motor-control status, while higher spasticity and pain were inversely related to achieved goals. The study is relevant because it links BoNT-A to individualized functional objectives, although it focuses primarily on upper-limb goal achievement rather than gait, balance or instrumental muscle mechanical properties [76].	**5**
24	**Increasing the Passive Range of Joint Motion in Stroke Patients Using Botulinum Toxin: The Role of Pain Relief** *Trompetto C.; Marinelli L.; Mori L.; Bragazzi N.; Maggi G.; Cotellessa F.; Puce L.; Vestito L.; Molteni F.; Gasperini G.; Farina N.; Bissolotti L.; Sciarrini F.; Millevolte M.; Balestrieri F.; Restivo D.A.; Chisari C.; Santamato A.; Del Felice A.; Manganotti P.; Serrati C.; Curra A.* **Design/type:** Retrospective clinical study.	**Sample:** Post-stroke patients treated for elbow and/or finger-flexor spasticity; the extracted report described 48 elbow-flexor cases and 64 finger-flexor cases, including a subgroup with reduced passive range of motion. **Follow-up:** Short-term pre/post treatment comparison; exact post-injection interval should be verified from the full text.	**Intervention:** IncobotulinumtoxinA injection with physiotherapy; no randomized comparator. **Target:** Elbow flexors and finger flexors.	Passive range of motion (p-ROM), spasticity/tone, pathological posture and pain intensity.	BoNT-A was associated with reduced tone, improved pathological posture and pain relief in treated flexor patterns. Passive range of motion improved more clearly in finger flexors than in elbow flexors, suggesting that apparent ROM gains may be partly mediated by pain reduction rather than reversal of fixed soft-tissue shortening. This is relevant to the distinction between spasticity, contracture, pain-limited movement and passive stiffness [77].	**5**
25	**Does the Diffusion Profile Differ Between Botulinum Toxin Type A Formulations? Implications for the Management of Post-Stroke Spasticity** *Picelli A.; Tamburin S.; Di Censo R.; Smania N.; Filippetti M.* **Design/type:** Technical/narrative review of BoNT-A formulation diffusion.	**Sample:** NA. **Follow-up:** NA.	**Intervention:** Discussion of diffusion profiles of BoNT-A formulations and implications for clinical management. **Target:** NA for a single target-muscle protocol; relevant to muscles adjacent to the intended injection site.	NA for direct patient outcomes in this table.	The paper is relevant for explaining why injection precision, dose distribution and unintended spread may influence efficacy and weakness. It provides mechanistic and procedural context, but it should not be summarized as a primary clinical outcome study [78].	**4**
26	**Estimating the cost consequence of the early use of botulinum toxin in post-stroke spasticity: Secondary analysis of a randomised controlled trial** *Lindsay C.; Humphreys I.; Phillips C.; Pandyan A.* **Design/type:** Secondary economic analysis of a randomized controlled trial.	**Sample:** Participants derived from the parent randomized trial; exact sample size should be verified from the full text. **Follow-up:** Follow-up according to the parent trial; exact duration should be verified from the full text.	**Intervention:** Early BoNT use in patients at risk of post-stroke spasticity and contracture-related costs. **Target:** Spastic limb muscles at risk of contracture; exact target muscles should be verified from the parent trial.	Cost consequences and contracture-related economic outcomes; clinical measures inherited from the parent trial.	The study supports the argument that earlier BoNT-A treatment may have economic value by reducing contracture-related consequences. It is useful for health-economic context but should not be used as a primary clinical efficacy study unless the parent-trial clinical details are extracted separately [79].	**4**
27	**Barriers to Long-Term Adherence in Botulinum Toxin Therapy for Post-Stroke Spasticity: Insights and Implications from a Single-Center Study in North Italy** *Cecchella E.; Bragazzi N.L.; Cotellessa F.; Campanella W.; Puce L.; Marinelli L.; Curra A.; Schenone C.; Mori L.; Trompetto C.* **Design/type:** Retrospective observational single-center adherence study.	**Sample:** 106 post-stroke spasticity patients treated between January 2013 and December 2023. **Follow-up:** Long-term real-world follow-up across the 2013–2023 treatment period; median number of injections and treatment duration were analyzed.	**Intervention:** IncobotulinumtoxinA treatment pathway and adherence/discontinuation analysis; no randomized comparator. **Target:** Individualized target muscles; exact injection muscles are not central to the adherence analysis.	Treatment continuation/discontinuation, number of injections, reasons for discontinuation, Kaplan–Meier survival analysis and Cox regression predictors.	Most patients continued treatment, but a substantial minority discontinued. Logistical barriers and comorbidities were prominent reasons for discontinuation, and logistical barriers were strongly associated with dropout risk. The study is important for implementation and follow-up planning but does not evaluate direct biomechanical, gait or balance outcomes [80].	**4**
28	**The Elias University Hospital Approach: A Visual Guide to Ultrasound-Guided Botulinum Toxin Injection in Spasticity. Part III—Proximal Lower Limb Muscles** *Popescu M.N.; Capet C.; Beiu C.; Berteanu M.* **Design/type:** Technical/visual guide; contextual injection-method paper.	**Sample:** NA. **Follow-up:** NA.	**Intervention:** Ultrasound-guided BoNT injection technique for proximal lower-limb muscles. **Target:** Proximal lower-limb muscles relevant to spastic gait and posture disorders.	NA; procedural anatomical guidance rather than clinical outcome measurement.	This article is relevant for anatomical targeting and ultrasound-guided injection planning in proximal lower-limb spasticity. It should be used as procedural support only, not as evidence of gait, balance or muscle-property changes after BoNT-A [81].	**4**
29	**Comparative efficacy and acceptability of non-invasive neuromodulation technologies and botulinum toxin injections for post-stroke spasticity and motor function: a network meta-analysis of randomised controlled trials** *Huang J.; Bao C.; Chen Y.; Zhu W.; Zhang K.; Liu C.; Tang C.* **Design/type:** Network meta-analysis of randomized controlled trials; secondary evidence.	**Sample:** NA for a single patient cohort; includes multiple RCTs. **Follow-up:** Variable across included RCTs.	**Intervention:** Comparison of non-invasive neuromodulation technologies and BoNT injections for post-stroke spasticity and motor function. **Target:** Upper and/or lower limb spasticity depending on included trials.	Spasticity and motor-function outcomes across included RCTs; exact measures vary.	The article provides comparative context for BoNT-A versus non-invasive neuromodulation technologies. It can support background discussion of treatment alternatives, but because it is a network meta-analysis, it should not be treated as a primary study in the structured extraction table [82].	**4**

**Table 3:** Brief description of the three studies included in our systematic literature review by manual searching.

No.	Study/Design	Sample & Follow-Up	Intervention & Target Muscles	Outcome Measures	Main Findings/Relevance
1	**Early Botulinum Toxin Type A Injection May Improve Motor Recovery in Patients with Post-Stroke Spasticity: A Secondary Analysis from a Longitudinal Cohort Study** *Alessandro Picelli, Andrea Santamato, Michela Cosma, Alessio Baricich, Carmelo Chisari, Marzia Millevolte, Cristina Del Prete, Ilenia Mazzù, Rita Di Censo, Nicola Smania, Mirko Filippetti* **Design/type:** Secondary analysis of a multicenter, open-label, longitudinal cohort study.	**Sample:** 83 BoNT/A-naïve patients with post-stroke spasticity; early treatment ≤ 90 days vs. late treatment > 90 days after stroke onset. **Follow-up:** Baseline, 4, 12 and 24 weeks post-injection.	**Intervention:** Individualized BoNT/A injection combined with conventional rehabilitation. **Target:** Upper- and/or lower-limb muscles according to spasticity pattern; commonly injected muscles included finger/wrist flexors and biceps brachii, and lower-limb muscles such as gastrocnemius medialis/lateralis, soleus and tibialis posterior.	MAS as primary muscle-tone outcome; Motricity Index, Fugl–Meyer Assessment and modified Rankin Scale as secondary outcomes.	Early BoNT/A administration was associated with greater reduction in spasticity at early follow-up and with faster motor recovery trajectories, especially in initially more impaired patients. The study supports early neuromodulation within rehabilitation, but it did not include direct objective muscle-property assessment such as myotonometry/elastography or dedicated stabilometric balance evaluation [83].
2	**The Effect of Short-Term Treatment with Botulinum Toxin A on Muscle Stiffness in Stroke Patients: An Exploratory Study** *Jelena Simic, Kristin Østlie, Fin Biering-Sørensen, Bo Biering-Sørensen, Derek John Curtis, Arve Opheim* **Design/type:** Prospective cohort exploratory study.	**Sample:** 25 first-stroke survivors with disabling spasticity and reduced walking function. **Follow-up:** Baseline, 6 weeks and 3 months after BoNT-A treatment.	**Intervention:** BoNT-A administered into clinically relevant calf muscles. **Target:** Calf muscles; medial gastrocnemius stiffness was measured on the affected and unaffected sides.	Shear-wave elastography muscle stiffness; MAS; active and passive ankle dorsiflexion; 10 m walk test; Goal-Attainment Scale.	Shear-wave elastography detected a significant reduction in affected-side muscle stiffness at 6 weeks, but the effect was not maintained at 3 months; no comparable change was observed in the unaffected limb. The study is directly relevant because it uses an objective, non-invasive muscle-property tool after BoNT-A, although the relationship between stiffness changes, walking function and goal achievement requires further longitudinal confirmation [84].
3	**Efficacy on Gait and Posture Control After Botulinum Toxin A Injection for Lower-Limb Spasticity Treatment After Stroke: A Randomized Controlled Trial** *Hui-xian Yu, Si-hao Liu, Zhao-xia Wang, Chang-bin Liu, Pei Dai, Da-wei Zang* **Design/type:** Randomized controlled trial with single-blinded assessments.	**Sample:** 46 patients with post-stroke hemiplegic gait randomized to experimental and control groups; 43 completed follow-up (21 experimental, 22 control). **Follow-up:** 0, 1, 4 and 12 weeks after treatment.	**Intervention:** Electrical-stimulation-guided BoNT-A plus routine rehabilitation versus routine rehabilitation/control care. **Target:** Lower-limb muscles selected according to gait pattern: quadriceps femoris, gastrocnemius, tibialis posterior, flexor hallucis longus, flexor digitorum longus, flexor digitorum brevis and flexor hallucis brevis.	Lower-limb MAS, lower-limb Fugl–Meyer Assessment, 10 m walk test, timed Up and Go test, gait analysis and dynamic plantar-pressure analysis.	Compared with control care, BoNT-A was associated with better lower-limb motor function and spasticity outcomes, improved stride length and walking speed, shorter TUGT time, and improved plantar-pressure/postural-control parameters across follow-up. This paper is highly relevant for mobility and balance outcomes after lower-limb BoNT-A, although it does not assess muscle stiffness through myotonometry or elastography [35].

## Data Availability

Not applicable.

## Abbreviations

The following abbreviations are used in this manuscript:

BoNT-A	Botulinum Toxin Type A
COP	Center-of-Pressure
PRISMA	Preferred Reporting Items for Systematic Reviews and Meta-Analyses
PICO	Patient/Population, Intervention, Comparison, and Outcome
PMC	PubMed Central
PEDro	Physiotherapy Evidence Database
ISI	Institute of Scientific Information
6 MWT	6 Min Walk Test
MD	Mean Difference
CI	Confidence Intervals
RCT	Randomized Controlled Trial
PROSPERO	International Prospective Register of Systematic Reviews
ESWT	Extracorporeal Shock Wave Therapy
GO-FAST	Goal-Oriented Facilitated Approach to Spasticity Treatment
ROM	Range of Motion
